# Barriers and enablers to effective interprofessional teamwork in the operating room: A qualitative study using the Theoretical Domains Framework

**DOI:** 10.1371/journal.pone.0249576

**Published:** 2021-04-22

**Authors:** Cole Etherington, Joseph K. Burns, Simon Kitto, Jamie C. Brehaut, Meghan Britton, Sukhbir Singh, Sylvain Boet

**Affiliations:** 1 Department of Anaesthesiology and Pain Medicine, University of Ottawa, Ottawa, Ontario, Canada; 2 Clinical Epidemiology Program, The Ottawa Hospital Research Institute, Ottawa, Ontario, Canada; 3 Department of Innovation in Medical Education, University of Ottawa, Ottawa, Ontario, Canada; 4 School of Epidemiology & Public Health, University of Ottawa, Ottawa, Ontario, Canada; 5 Main Operating Room, The Ottawa Hospital, Ottawa, Ontario, Canada; 6 Department of Obstetrics & Gynecology, University of Ottawa, Ottawa, Ontario, Canada; 7 Francophone Affairs, Faculty of Medicine, University of Ottawa, Ottawa, Ontario, Canada; 8 Institut du Savoir Montfort, Ottawa, Ontario, Canada; 9 Faculty of Education, University of Ottawa, Ottawa, Ontario, Canada; Hong Kong Polytechnic University, HONG KONG

## Abstract

**Background:**

Effective teamwork is critical for safe, high-quality care in the operating room (OR); however, teamwork interventions have not consistently resulted in the expected gains for patient safety or surgical culture. In order to optimize OR teamwork in a targeted and evidence-based manner, it is first necessary to conduct a comprehensive, theory-informed assessment of barriers and enablers from an interprofessional perspective.

**Methods:**

This qualitative study was informed by the Theoretical Domains Framework (TDF). Volunteer, purposive and snowball sampling were conducted primarily across four sites in Ontario, Canada and continued until saturation was reached. Interviews were recorded, transcribed, and de-identified. Directed content analysis was conducted in duplicate using the TDF as the initial coding framework. Codes were then refined whereby similar codes were grouped into larger categories of meaning within each TDF domain, resulting in a list of domain-specific barriers and enablers.

**Results:**

A total of 66 OR healthcare professionals participated in the study (19 Registered Nurses, two Registered Practical Nurses, 17 anaesthesiologists, 26 surgeons, two perfusionists). The most frequently identified teamwork enablers included people management, shared definition of teamwork, communication strategies, positive emotions, familiarity with team members, and alignment of teamwork with professional role. The most frequently identified teamwork barriers included others’ personalities, gender, hierarchies, resource issues, lack of knowledge of best practices for teamwork, negative emotions, conflicting norms and perceptions across professions, being unfamiliar with team members, and on-call/night shifts.

**Conclusions:**

We identified key factors influencing OR teamwork from an interprofessional perspective using a theoretically informed and systematic approach. Our findings reveal important targets for future interventions and may ultimately increase their effectiveness. Specifically, achieving optimal teamwork in the OR may require a multi-level intervention that addresses individual, team and systems-level factors with particular attention to complex social and professional hierarchies.

## Introduction

Effective teamwork is essential for safe, high-quality healthcare [1, 2]. It is particularly important in the operating room (OR), where professionals of different disciplines, educational backgrounds, and experiences must work interdependently in a dynamic, high-stakes environment [3–10]. Studies have documented that OR teamwork “failures” (i.e. suboptimal practice) are very common: some indicate that teamwork issues happen to various degrees in every surgery [6, 11, 12] while others observe the rate of suboptimal teamwork practices to be as high as 17.4 per hour [13]. The odds of surgical complications are approximately five times higher when interprofessional teamwork is ineffective [6].

Although many studies have explored various facets of OR teamwork [14–16], there has yet to be a systematic assessment of teamwork barriers and enablers that can directly inform behavior change interventions. Instead, there has remained a gap between observational studies of teamwork offering in-depth accounts of practices within a particular context and interventional studies that aim to improve teamwork. For example, most interventions involve team training to improve some element of teamwork (e.g. communication) [17], yet observational research indicates that teamwork is a complex phenomenon influenced by multiple factors across multiple levels (i.e. individual, team, organization) [14, 18]. A single-faceted strategy such as team training is unlikely to address the multilevel factors required for a substantial and sustainable improvement in OR teamwork as a whole. Not surprisingly, most teamwork interventions result in a limited effect on teamwork and associated outcomes [17]. In addition, there has not been any substantial reduction in patient safety events in recent years [19, 20].

The Theoretical Domains Framework (TDF) is a tool used in the field of implementation science to close the gap between research and practice. It was specifically developed to elicit determinants of clinical behaviour and to inform the design of behaviour change interventions [21, 22]. As a comprehensive framework comprised of key psychological theories and constructs, the TDF has been applied in a variety of healthcare settings to understand the factors driving current practice in order to change clinician behaviour [23–30]. Applying the TDF to behaviour (e.g. teamwork) allows its determinants (i.e. barriers and enablers) to be mapped to specific behaviour change techniques and modes of delivery [31, 32]. This approach increases the likelihood of influencing healthcare professional behaviour in a meaningful and clinically significant way [23, 32, 33]. This study therefore aimed to identify factors that facilitate or impede OR teamwork from an interprofessional perspective using the TDF in order to inform future evidence-based, actionable interventions.

## Methods

This study is reported in accordance with the Standards for Reporting Qualitative Research checklist [34]. Ethical approval was obtained from the Ottawa Health Science Network Research Ethics Board (#20170875; coordinating site) and the Unity Health Toronto Research Ethics Board (REB #18–396).

### Study design

We conducted a prospective, multicentre qualitative study using semi-structured interviews, either in person or over the telephone, with OR healthcare providers. Participant informed consent was obtained from each participant by the study interviewers (JB, NE). For interviews conducted in person, the interviewer obtained informed written consent. For interviews conducted over the telephone, verbal consent was documented by the interviewer on the participant master list.

### Setting and context

Recruitment primarily took place across six sites: one academic hospital located in Toronto, Canada and three academic hospitals located in Ottawa, Canada, one of which had three campuses. Each hospital performs a large volume of a wide range of surgical procedures, such as general, gynecological, trauma, pediatric, and cardiac surgeries, and both oncological and non-oncological surgeries. Participants at each site were invited to refer colleagues to the study as well, creating the possibility for representation of additional sites.

### Sample and recruitment

All healthcare professionals working in the OR were eligible to participate, including scrub and circulating nurses, anaesthesiologists, perfusionists, anaesthesia assistants, surgeons, and anaesthesia and surgical post-graduate trainees. Across the six primary recruitment sites, there were over 1000 eligible healthcare professionals. Healthcare professionals not part of the OR team at these or referred sites were not eligible to participate. We used volunteer, purposive, and snowball sampling to optimize recruitment [35]. An invitation to participate in the study was emailed to the perioperative departments of each participating hospital site. Individuals participated in the study on a “first come, first served” basis, unless data saturation had occurred within their particular professional group. Participants were also invited to refer their colleagues from outside their centres to the study. Purposive sampling was used to obtain representativeness among professional groups where it was observed necessary (e.g. to obtain both male and female nurses). Participants were offered a gift card for participation upon completing their interview. Representativeness of the sample is limited, however, to OR team members at academic hospitals in Ontario, Canada, as this is where our primary recruitment took place.

### Theoretical framework

A modified version of the Theoretical Domains Framework (TDF) [23, 36], guided interview guide development, data collection, and analysis. The TDF is a validated framework for ascertaining barriers and facilitators to behaviour change among healthcare professionals [23]. It consists of 14 theoretical domains (S1 Appendix) derived from 128 theoretical constructs from 33 theories of behaviour and behaviour change relevant to determinants of behaviour and intervention development in healthcare [23, 36]. The domains were derived through a systematic expert consensus process and provide a basis for understanding the broad set of factors that may influence behaviour. Although the TDF has been used to examine barriers and enablers to various behaviours across many healthcare settings [23], it has yet to be used to study interprofessional teams in the OR.

### Interview guide

The semi-structured interview guide (S2 Appendix) developed by the co-investigator team comprised of practicing OR clinicians from anaesthesia, nursing, and surgery as well as researchers with backgrounds in medical education, qualitative research, implementation science, psychology, and sociology. The interdisciplinary background of the team helped to ensure the interview guide was comprehensive through inclusion of anaesthesia, nursing, and surgical perspectives as well as educational, sociological, and psychological considerations.

The guide included open-ended questions based on the TDF domains along with additional questions to explore aspects of social identity (e.g. gender, age, ethnicity) and teamwork for a separate study. The questions covered experiences and perspectives of teamwork according to participants’ professional contexts and explored factors influencing teamwork at the individual and team level. We pilot tested the interview guide with two OR clinicians (one physician and one nurse) to provide feedback on clarity of questions and revised the wording of several questions accordingly. The guide was iteratively revised throughout the interview process to better capture new themes introduced by participants and to remove or rephrase questions participants had difficulty answering, as per best practices in qualitative research [37]. Examples of questions asked include “How would you define teamwork?” (*knowledge*); “As a [profession], is there anything that influences your approach to teamwork in the OR?” (*social/professional role and identity*); “Do your emotions every influence whether or not you engage in good teamwork in the OR?” (*emotions*); and “Would any other team member influence whether or not you engage in good teamwork in the OR?” (*social influences*).

### Interviewer training

Interviews were conducted in English by two research team members (JKB, NE) with no previous relationships to participants. The research team members were each trained in qualitative research methods and experienced in working within sensitive healthcare environments. Prior to conducting the interviews, the research team members familiarized themselves with the interview guide and conducted a practice interview. The interview guide was then further refined to facilitate conversation flow by adding several broad introductory questions (e.g. “What does teamwork mean to you?”) and re-ordering some of the other questions.

### Data collection

Interviews were scheduled with interested participants over a four-month period (January to April 2019). Demographic information was collected from all participants (e.g. profession, years of experience, age, sex) at the time of their interview. Interviews continued until data saturation was reached, which was defined as conducting three interviews within each major professional group (anaesthesia, nursing, surgery) without the emergence of any new themes (after a minimum of 10 interviews per group) [38]. The interviewers met regularly to discuss themes emerging from the interviews and assess whether data saturation had been met.

### Data analysis

Interviews were audio-recorded, transcribed, and de-identified. De-identified transcripts were imported into *NVivo 12* (QSR International, Doncaster, Australia) for analysis. Deductive directed content analysis was conducted by two research team members (JKB, NE) using the TDF as the coding framework. To establish consistency between coders, three interviews were first coded independently; after which the two coders met to review their coding, resolve discrepancies, and determine a coding strategy. All remaining interview transcripts were then divided equally between the two coders and coded using the agreed upon coding scheme. The coders met frequently to triangulate the data and maintain reliability, with 10% of the remaining interviews coded in duplicate.

After data were coded into the TDF domains, data units (i.e. several lines of text) were labelled with codes according to the key concept represented within the data unit by one coder (NE). These codes were refined whereby similar codes were grouped into larger themes within each TDF domain. Findings were summarized within and across each main professional group (i.e. nursing, anaesthesia, surgery) and verified by a second coder (JKB). TDF domains were classified as relevant to intraoperative teamwork (i.e., more likely to influence teamwork) based on: the frequency of specific beliefs across interviews (four or more clinicians identified the belief in their interview), the number of beliefs in each domain (more than two), the presence of conflicting beliefs signaling variation in beliefs and attitudes, and evidence of strong beliefs that could directly influence teamwork performance [23]. Based on all these factors, we concurrently established domain relevance.

## Results

### Participant characteristics

A total of 66 healthcare professionals who currently practice in the OR were interviewed (Table 1). Of these, 19 (29.9%) were Registered Nurses (15 female, 4 male), two (3%) were Registered Practical Nurses (1 female, 1 male), 17 (25.8%) were anaesthesiologists (9 female, 8 male), 26 (39.4%) were surgeons (10 female, 16 male), and two (3%) were perfusionists (1 female, 1 male). Approximately one third of participants (n = 23 [34.8%]) were anaesthesia or surgical trainees (i.e. residents or fellows). Most participants practiced in Ottawa (n = 55 [83.3%]). Among surgeons (n = 26), 54% reported general surgery as their specialty (n = 14). The median age of participants was 35 years (IQR = 29–43).

**Table 1:** Demographics of participants included in study (n = 66).

	N (%) or Median (IQR)
**Profession**	
Registered Nurse (RN)	19 (28.8%)
Surgeon (Post-graduate trainee)	14 (21.2%)
Surgeon (Attending)	12 (18.2%)
Anaesthesiologist (Post-graduate trainee)	9 (13.6%)
Anaesthesiologist (Attending)	8 (12.1%)
Perfusionist	2 (3.0%)
Registered Practical Nurse (RPN)	2 (3.0%)
**Surgical specialty** *	
General	14 (53.8%)
Orthopedic	3 (11.5%)
Urology	3 (11.5%)
Gynecology/obstetrics	1 (3.8%)
Neurosurgery	1 (3.8%)
Otolaryngology	1 (3.8%)
Plastic	1 (3.8%)
Thoracic	1 (3.8%)
Trauma	1 (3.8%)
**Hospital location**	
Toronto	9 (13.6%)
Ottawa	55 (83.3%)
Other	2 (3.1%)
**Age**	35 (29–43)
**Sex**	
Female	36 (54.5%)
Male	30 (45.5%)

*applies to surgery only (n = 26).

### Overview of barriers and enablers identified within relevant domains

**Fig 1:**
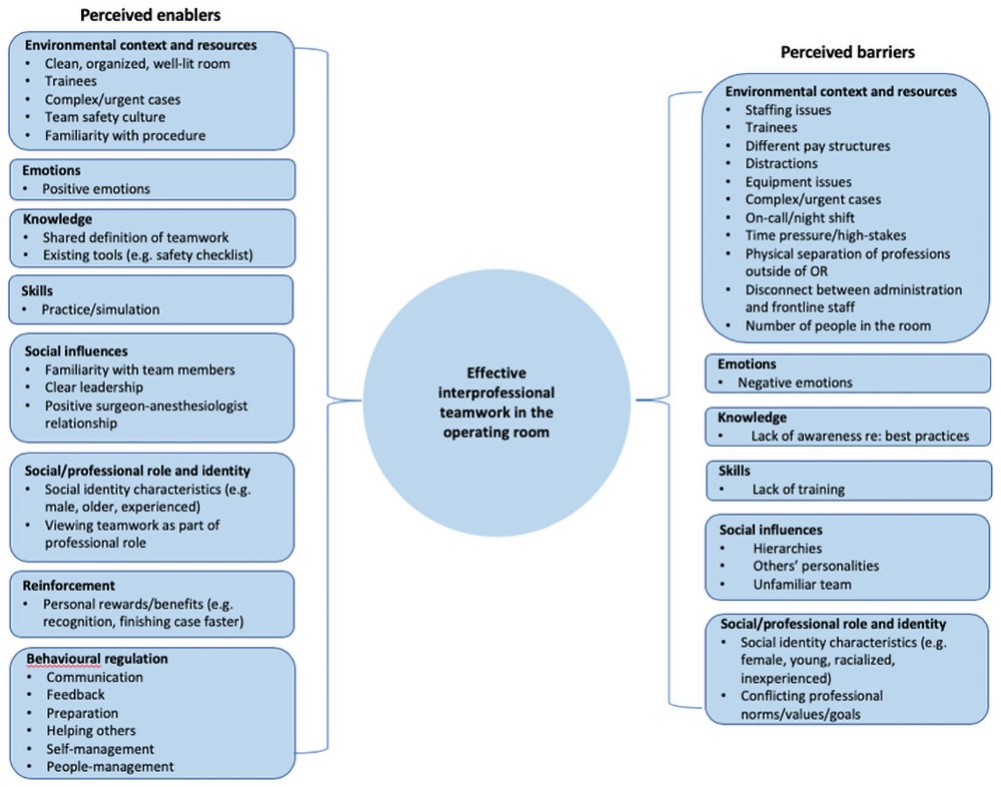
Barriers and enablers to interprofessional teamwork in the OR reported by participants (n = 66).

Barriers and enablers were identified across eight relevant domains (*behavioural regulation*; *emotions*; *environmental context and resources*; *knowledge*; *reinforcement*; *skills*; *social influences*; *social/professional role and identity*). Fig 1 summarizes all of the barriers and enablers identified by participants across all relevant domains, with further details provided in S3 Appendix. The most commonly identified behavioural domains and themes are highlighted in Table 2.

**Table 2:** Most frequently identified themes across relevant TDF domains*.

Domain	Theme	Frequency of theme n participants (%)			
		Anaesthesia (n = 17)	Nursing (n = 23)^a^	Surgery (n = 26)	Total across professions (n = 66)
Behavioural regulation	Communication practices or strategies (+)	12 (71.0%)	17 (73.9%)	18 (69.2%)	47 (71.2%)
	People management (+)	17 (100%)	10 (43.5%)	26 (100%)	65 (98.5%)
Emotions	Positive/negative emotions (+ -)	15 (88.2%)	18 (78.3%)	22 (84.6%)	55 (83.3%)
Environmental context and resources	Severity or type of case (+ -)	12 (70.6%)	5 (21.7%)	23 (88.5%)	40 (60.6%)
	On-call/night shift (-)	10 (58.8%)	9 (39.1%)	15 (57.7%)	34 (51.5%)
	Resource-related challenges (-)	8 (47.1%)	23 (100%)	22 (84.6%)	53 (80.3%)
Knowledge	Lack of awareness regarding best practices (-)	10 (58.8%)	15 (65.2%)	14 (53.8%)	39 (59.1%)
	Shared definition of teamwork (+)	15 (88.2%)	15 (65.2%)	24 (92.3%)	54 (81.8%)
Social influences	(Un)Familiarity with team members (+ -)	10 (58.8%)	10 (43.5%)	18 (69.2%)	38 (57.6%)
	Hierarchies (-)	13 (76.5%)	17 (73.9%)	24 (92.3%)	54 (81.8%)
	Others’ personalities (-)	14 (82.4%)	22 (95.7%)	24 (92.3%)	60 (90.9%)
Social/professional role and identity	Gender (-)	13 (76.5%)	21 (91.3%)	25 (96.2%)	59 (89.4%)
	Teamwork is part of professional role (+)	10 (58.8%)	13 (56.5%)	13 (50.0%)	36 (54.5%)
	Conflicting professional norms/values/goals/perceptions (-)	10 (58.8%)	9 (39.1%)	18 (69.2%)	37 (56.1%)

*Theme identified by 50% or more of all participants and/or 50% or more of one professional group.

^a^ Perfusionists (n = 2) were grouped with RNs (n = 19) and RPNs (n = 2) for the purposes of this summary.

(+) = enabler; (-) = barrier; (+ -) = viewed as enabler by some participants and barrier by others.

Cell frequency colour legend: darker to lighter shading = higher to lower frequencies

### Most commonly identified themes within relevant domains

Key themes, domains and illustrative quotes are summarized in Table 3 and discussed below, in order of most to least frequently discussed.

**Table 3:** Most commonly identified key themes and illustrative quotes.

Theme (domain)	Illustrative quote
(+) People management (behavioural regulation)	“I, like some of my colleagues, probably resent a little bit being called, “Hey, anaesthesia,” you know? I have a name too and I think people just respond better to that”—Anaesthesiologist 1.
(-) Others’ personalities (social influences)	“…I mean, it’s hard to take your personality out of it, right? There’s one or two nurses, for example, who really rub me the wrong way. We just… we have personalities that, you know, we’d never be friends outside of work. Let’s just say that.”—Surgeon 22
(-) Gender and other social identity factors (social/professional role and identity)	“…you know, strong female personalities are often perceived differently than strong male personalities. You know, whereas I think a man who has a really strong personality who’s, you know, maybe a little bit more rigid and not quite as flexible… which is often viewed as, you know, being themselves and, you know, they’re kind of a tough guy but they’re a leader, whereas I think where women demonstrate some of those same behaviours, they’re thought of as bitchy or difficult and it’s just that same behaviour be framed in a different context. So, I think in some ways it can be a little bit harder to be a woman and be respected as a leader without coming across as being difficult.”–Surgeon 9 “Being Asian, you know, being an Asian girl, and I look young. Definitely, I’m not treated the same as other people… I just… so I know what I’m walking into, and I’m not going to get upset about it, because it’s just a fact now that’s what I’m going to get most of the time…”–RN 1
(+ -) Emotions (emotion)	“I remember working with one of the surgeons who was just very demeaning and very demanding… it’s hard to put into words but I found that that was really hard because everyone was kind of scared all the time, and tiptoeing around… that’s not great because people are kind of in an environment of fear and that’s not the best for the patient, right?”–Surgeon 2
(-) Hierarchies (social influences; environmental context and resources)	“…the stereotypical situation is you have a medical student or a PGY1 who’s like, five minutes into their training, and they sort of walk in like, ‘I’m the doctor, you’re the nurse!’ And you have a nurse that’s been in practice for 30 years and is fantastic and really knows their stuff. And there’s a bit of a power struggle there because, I mean, technically, the doctor is, quote unquote, ‘in charge’. But, that being said, that nurse knows a whole lot more. And so, when you have people that feel the need to exert their expertise or sort of show their position in a social hierarchy, I think that makes the team dynamic much more difficult.”–Surgeon 18 “[our breakroom is] more around the OR, but I find that we don’t really have one common space where we all hang out before or after or in-between ORs… in general anyways. There’s the surgeon’s area and then there’s a separate nursing area. I don’t necessarily know why there is or needs to be that distinction. And where does housekeeping hang out? You know what I mean?”–Surgeon 5
(-) Resource-related challenges (environmental context and resources)	“So lately we have a lot of equipment issues, for example, so when the equipment’s missing, the surgeon’s pissed because they don’t have the right things they need and who are yelling at you, “hey, Barbara, where’s the blah blah.” And it’s not Barbara’s fault that she doesn’t have it but… so yeah… You can have a great team that’s really working hard and really rolling well, the atmosphere’s great but if there’s a whole bunch of equipment problems, that can have a huge impact on the day and people’s morale and then all of a sudden you see the teamwork getting a little shakier and a little more rocky and all the pieces aren’t playing quite as well.”–Surgeon 6 “They [surgeons] often think it’s more related to incompetency but they also don’t realize that, at least in my current place right now, our training is very bad. Our orientation is really short because we’re short staffed, so they’re cutting our training in half. You know, it takes time to be familiar with a procedure. So, if you’re new in the OR it’s going to take a number of years. So, I think for them to be empathetic and realizing that and not raising their voice and understanding, okay well this is what I need next, then it’s fine, just communicate that. You know, all you got to do is just communicate it.”–RN 5
(+) Shared definition of teamwork (knowledge)	“I would [define teamwork as] everyone working towards a common goal and a common goal being a good outcome for the patient.”–Surgeon 7
(+) Communication practices or strategies (behavioural regulation)	“I like to ask a lot of questions… So, when I see people, I like to clarify things. I like to repeat things to make sure that there’s no errors. And I like to include people when decisions are made. And sometimes you have to go out of your way to do some of those things.”–RN 15
(+ -) Severity or type of case (environmental context and resources)	“Yes, actually it’s funny. When it is an emergency situation, I find the communication’s a lot better because I think the surgeon realizes we don’t know exactly what they need. And, for some reason, for elective cases they have this impression that we know exactly what they want at all times. It’s like they’ve written down like a detailed instruction booklet for the case and we’re kind of confused and… I mean I think there needs to be more current communication each day. Like but for emergency situations, I think it’s actually a lot better.”–RN 6
(-) Lack of awareness of best practices (knowledge)	“… sorry I’m not really aware of what’s best practice.”–RN 17
(+ -) Familiarity (social influences)	“I would add a little insight into saying that I think it’s easy now because I’ve been here for six years and I know everybody. And I work at the [campus name], very rarely I take call at the [campus name] and there I actually find it a little bit challenging because I don’t know people. People don’t know me. So it’s hard when you don’t… and people kind of inherently trust you as the surgeon but it’s not really the same when they don’t know you, you know, or… so I think in those moments it’s actually quite challenging when you don’t know your team members quite as well.”–Surgeon 1
(-) Conflicting professional norms/values/goals/perceptions (social/professional role and identity)	“I think everyone has the same global objective, but people might have different attributes of what they think makes an effective team. But that might almost be like the discrepancies in what their individual agendas are. Like, for example, like a surgeon might be really concerned about like doing something a particular way but maybe the anaesthetist is more worried about their time under GA being shorter. Overall both of them care about the patient’s well-being but because they have different specific agendas they might not share like the same focus as their own. Things could kind of misalign there.”–Surgeon 4
(+) Teamwork as part of professional role (social/professional role and identity)	“I think because it’s just so ingrained in you as a nurse in general, like even from your training in nursing, like in university or college, it’s ingrained in you that, you know, nurses are teams, you work as a team..”–RN 9
(-) On call/night shift (environmental context and resources)	“…on the day you’ve been up all night, and still working the full next day, you’re not as enthused with teamwork, you’re more just trying to survive through the day and then go”—Surgeon 13

(+) = enabler; (-) = barrier; (+ -) = viewed as enabler by some participants and barrier by others.

#### People management (n = 65 [98.5%]; *behavioural regulation*)

The most common theme identified across participant responses was the role of “people management” in facilitating teamwork. Managing the people in the room was reported by 100% of anaesthesiologists and surgeons (n = 17, n = 26) to facilitate teamwork compared to less than 50% of nursing staff (n = 10). Participants identified a variety of strategies that they would often use in this regard, ranging from simply “being nice” to others to actively building relationships. As part of these strategies, participants emphasized the importance of using everyone’s names (e.g. writing down everyone’s name on a piece of paper in order to remember it).

#### Others’ “personalities” (n = 60 [90.9%]; *social influences*)

The influence of others’ “personalities” was frequently cited by participants as a barrier to teamwork in the OR. Participants acknowledged that there are certain team members who do not always work well together on a personal level. Faced with these situations, many participants expressed that they would try to adjust their behaviour or approach in the OR according to the “personalities” on the team that day. This could be challenging, however, when working with “difficult personalities” and participants expressed that there are certain individuals who are “just difficult to work with” (Anaesthesiologist 8) and who “don’t play nice with others” (Anaesthesiologist 15). Personality was also spoken about as a factor separate from skill level or professional role, which “spills over into every aspect of care” (Perfusionist 2).

#### Gender and other social identity factors (n = 59 [89.4%]; *social/professional role and identity*)

The third most common theme highlighted by participants was gender. The role of gender in the OR appeared to be particularly recognized among surgeons, with all but one discussing the influence of their own gender on teamwork or their observations about how others experience challenges related to gender (n = 25 [96.2%]). Several participants also acknowledged the “privilege” they experienced as white male physicians, whereby they often reported that they had an “easier” time in the OR in terms of obtaining respect, demonstrating leadership, and maintaining positive interactions. Conversely, team dynamics could be different when a female physician asserts leadership, and this was recognized by both male and female physicians. Many female participants also expressed how other elements of their social identity intersected with gender to shape their experiences in the OR (e.g. being female and Asian). Based on their social identity characteristics, participants expressed that they were often perceived by others as less competent and this placed strain on relationships within and across professions. Frequently, communication practices, perceptions of leadership, and acts of followership were reported by participants to vary depending on the social identities of the individuals in the room.

#### Emotions (n = 55 [83.3%]; *emotions*)

Emotions were described as both potential barriers and enablers of teamwork. In particular, several negative emotions associated with fear were noted to impede teamwork. Among the emotions discussed by participants, stress, being in a “bad mood”, or feeling scared of others, were indicated as barriers to teamwork while positive emotions were viewed as enablers of effective teamwork by participants. Participants also noted that the actions of others could influence their own emotions. One Registered Nurse shared that if a surgeon was yelling at them, it would cause them to feel afraid and subsequently withdraw from effective teamwork behaviours. Of note, 18 (27.3%) participants explicitly mentioned feeling scared, fearful, or intimidated with regard to interpersonal interactions in the OR, and this was primarily in reference to the dynamic between nurses and surgeons or between surgical residents and staff surgeons. Overall, these emotions were felt to be detrimental to patient care.

#### Hierarchies (n = 54 [81.8%]; *social influences; environmental context and resources*)

Participants described how various social hierarchies could affect teamwork, including conflicts between professional status and years of experience, such as a first-year resident challenging a nurse with 30 years of experience. Related to hierarchies, participants discussed how physical separation of professions outside of the OR (e.g. having separate lounges) further impacted interprofessional relationships and could reproduce interprofessional distinctions.

Participants also identified that hierarchies could be maintained within professional groups. One example was provided by an RN who described a new checklist introduced by management that “totally segregated” the nursing team by specifying “lead nurse, nurse number 2 and then RPN… [with] rules for each person.” (RN 6). Different meetings between different nursing and support team members (e.g. RNs, RPNs, orderlies) were also reported to cause divisions.

#### Resource-related challenges (n = 53 [80%]; *environmental context and resources*)

Resources, such as staffing and equipment issues, were identified by participants as barriers to teamwork. Notably, 100% of nursing staff (n = 23) and 85% of surgeons (n = 22) considered resource-related challenges to be a barrier to teamwork compared to 47% of anaesthesiologists (n = 8). Many tensions between nursing staff and surgeons were attributed by both groups to these issues.

#### Shared definition of teamwork (n = 54 [81.8%]; *knowledge*)

A key enabler for teamwork identified from participant responses was that most team members defined teamwork in the same way. Specifically, teamwork as spoken about as “working toward a common goal”, with the common goal being patient safety or a good outcome for the patient.

#### Communication practices or strategies (n = 47 [71.2%]; *behavioural regulation*)

Participants discussed a wide variety of communication practices or strategies which they used to facilitate teamwork. Examples included: asking questions, explaining actions out loud, expressing concerns in advance, including all team members in communications, speaking loudly, and calling for a pause or time-out.

#### Severity or type of case (n = 40 [60.6%]; *environmental context and resources*)

Participants highlighted clinical acuity as influencing teamwork, but there was variation in whether this was viewed as a barrier or enabler to teamwork. Some participants reported that teamwork improved during emergent cases, while others indicated that teamwork deteriorated with heightened urgency.

#### Lack of awareness of best practices (n = 39 [59.1%]; *knowledge*)

Nearly 60 percent of participants reported that they were not aware of any best practices for teamwork in the OR. Participants reported that teamwork “was not emphasized at any point” (Perfusionist 1) during their training and that they had not experienced anything “structured or formulated” (Surgeon 5). Teamwork was also spoken of as learned “on the job” and from mentors, rather than as a trainee. Participants expressed the desire for continuing professional education related to teamwork that would specifically bring the different OR professions together. As one surgeon explained, “you never really know” how you are perceived by others, and it is important to have feedback from other team members in addition to having simulation sessions with “the whole team practicing”.

#### Familiarity (n = 38 [57.6%]; *social influences*)

For effective teamwork to occur, over half of participants cited the importance of knowing other team members. Participants reported challenges when working in an OR with unfamiliar team members and revealed that there are different levels of trust depending on whether team members know each other or not.

#### Conflicting professional norms/values/goals/perceptions (n = 37 [56.1%]; *social/professional role and identity*)

Participants discussed how different professional socialization experiences and structures could pose challenges for teamwork. For example, “what would be leadership from a nursing perspective would be very different than leadership from a physician perspective, based on their roles and their training.” (Anaesthesiologist 2). Participants also acknowledged that there are “conflicting benefits” for each sub-team, such as the pressure for surgeons to move on to the next case while “from a union perspective” nurses are entitled to “breaks and lunches”, and that can put a case on hold (Surgeon 4).

#### Teamwork as part of professional role (n = 36 [54.5%]; *social/professional role and identity*)

Many participants reported that they viewed teamwork as part of their professional role. Surgeons reported that teamwork was important to them because they are “leaders within the room” whereas anaesthesiologists viewed teamwork as important based on their simulation and crisis resource management backgrounds. Nurses reported that “you cannot really do your entire job without having your team with you and guiding you and helping you” (RN 17).

#### On call/night shift (n = 34 [51.5%]; *environmental context and resources*)

Many participants viewed being on call or working a night shift as challenging for teamwork Participants expressed that the dynamic changes between team members during these shifts, where the focus becomes more on getting through the case rather than trying to be a good team member.

### Key themes in less relevant domains

Most participants reported that they made a conscious effort to engage in good teamwork (*intentions*) and that effective teamwork promoted positive outcomes for both patients (e.g. reduced complications) and clinicians (e.g. job satisfaction) (*beliefs about consequences*). Participants also generally viewed teamwork as important or desirable (*goals*) and reported that teamwork was something they put effort into and eventually became automatic over time (*memory*, *attention and decision processes*). The domains of optimism and beliefs about capabilities were not observed among participant responses.

## Discussion

This study identified barriers and enablers to effective interprofessional teamwork in the OR based on theoretically-informed interviews with a large sample of practicing OR healthcare professionals across several sites and specialties. Specifically, we obtained an interprofessional perspective of teamwork for surgical patient safety, which is important for taking a comprehensive approach to improving performance and outcomes in the OR. This sets our work apart from other studies of OR teamwork, which have been largely atheoretical, providing broad observations or suggestions for improving teamwork rather than specific and actionable information [14, 18].

In this study, personality conflicts across the professions were often cited as a barrier to teamwork and participants acknowledged the difficulty of working with individuals that they would not associate with outside of the OR. Although personality conflicts were discussed within professions, they were most apparent across professions, with one group perceiving another as “difficult” (e.g., surgeon-nurse conflicts). This finding is consistent with other studies showing discrepancies in perceptions of teamwork quality and team members’ roles among participants of different professions [39–42]. We also found that despite sharing a common overall definition of teamwork, participants often revealed how the norms and structures of their individual professions conflicted, straining interprofessional relationships in the OR, and posing a risk to patient safety. It may be that while OR clinicians agree on the concept of “teamwork”, the concept of “team” may be different. As Frasier and colleagues found, different OR professions offer a different definition of who they considered to be on their “team” for a given operation [43]. For example, nurses considered other nurses and technologists to be on their team and anaesthesiologists considers anyone involved in the provision of anaesthetic care to be on their team [43]. Consequently, a key challenge with OR teamwork may be its conceptualization as profession-specific rather than truly interprofessional.

Elements of power and hierarchy appeared to be at the centre of these issues, and participants often referred to the challenges associated with their position within the OR hierarchy. It was evident that profession is just one aspect of status in the OR, as participants explicitly discussed how various social factors (e.g., gender, profession, level of experience) worked together to shape perceptions and experiences of teamwork in the OR. Consequently, teamwork interventions may need to consider the simultaneous influence of multiple hierarchies rather than solely addressing interprofessional relations. A recent systematic review of interventions to improve OR culture reported that improvement strategies could be categorized as briefings/debriefings, team-building exercises, educational campaigns, and checklists [44]. Addressing social hierarchies, such as those related to gender or race, was not described in any of these intervention strategies. This may help to explain why there have been only moderate gains in patient safety in recent years despite the proliferation of teamwork interventions [45–48] given the significance of several types of hierarchies reported by participants in our study. Promoting inclusive leadership from an equity and diversity perspective [49] may therefore be an important consideration for future teamwork interventions.

The physical separation of professions outside of the OR was pointed out by several participants in our study. Familiarity was also frequently discussed by participants as critical for effective teamwork and has been reported on in other studies. For example, Reeves *et al*. found that, often in the perioperative environment, hierarchy and separateness between physicians and nurses is “compounded by their spending periods of time in separate spaces completing profession specific tasks or engaging in conversations with members from their own professional group” [50]. Others noted that relationships between healthcare providers, however, can change in different times and places [51]. For example, evidence shows communication patterns between physicians and nurses varies by where it takes place and is often more effective in more casual areas [52]. Therefore, one way to reduce the complex and intersecting hierarchies within the OR environment is to increase familiarity with team members [53]. An interprofessional lounge may be one component of a future intervention which could allow healthcare providers of different professions to interact with each other more frequently outside of case-related group tasks in the OR, facilitating a collaborative team culture [54]. Creating a collaborative culture is not just about exchanging information, but also fostering collaborative interprofessional working relationships [53]. This is often best accomplished in more “neutral” areas, such as an interprofessional lounge, where hierarchy can be alleviated as providers engage in collegial, casual social interactions [55]. Overall, putting in place organizational structures that require interprofessional interaction and reduced hierarchy, where providers can get to know each other “as people”, is a recommended best practice for teamwork in healthcare organizations [56] resulting in better patient care [57] and improved well-being for staff [58, 59]. In fact, a recent experimental study found that once clinicians were taken out of the workplace and put into a controlled setting, professional tribalism, hierarchical and stereotyping behaviours largely dissolved [60]. Yet, previous teamwork interventions for the OR have often overlooked the fundamental aspects of building collaborative relationships across social and professional and boundaries to overcome unconscious biases and professional silos [59, 61].

Along with increasing familiarity among team members, it may also be valuable to teach team members strategies for recognizing and challenging unconscious biases related to gender, ethnicity, and additional social identity factors [62]. This could be accomplished through trainee education and continuing professional development curricula [62]. Teaching team members specific strategies to assist individuals in speaking up or challenging authority when needed [63], along with conflict and emotion-management techniques [64], could also be valuable. It is important to note, however, that whether individuals feel able to use these strategies and skills may depend on their social position (e.g. gender, level of experience, profession). Future research may wish to examine whether intervention effectiveness varies by these characteristics. In any case, it is clear that addressing multiple aspects of power and hierarchy will be important to the success of any teamwork intervention.

Quantitative research suggests that equipment-related issues are correlated with higher stress and lower teamwork, particularly for nurses [65]. Equipment issues were particularly relevant to nursing staff and surgeons in our study, further supporting the relevance of these issues to interprofessional teamwork. Participants in our study also revealed that resource-related challenges (e.g. equipment and staffing availability) could further create tension between professional groups. These system-level factors should be considered in intervention development in order to promote sustainability. Even if teamwork practices can be improved and hierarchies reduced among OR teams, teamwork issues may be likely to still arise if staffing and equipment needs are not addressed.

A lack of knowledge and training regarding best practices for teamwork was identified as a barrier to teamwork across professions. To overcome this barrier, it may be useful to create a teamwork protocol, drawing upon strategies identified by OR team members. For example, participants in this study identified numerous strategies they used to facilitate communication, to manage their own behaviour, and to manage interactions with others. Of course, it is important to note some professional variation was observed regarding people management, whereby physicians appeared to engage in this strategy more frequently than nurses. This may reflect perceived differences in role, power, and influence among these two groups [41]. Once again, a teamwork protocol may help to overcome these discrepancies and empower all team members to engage in specific behaviours and actions to facilitate teamwork and patient safety. In any case, enhancing knowledge and training regarding best teamwork practices may also help to reduce some of the other barriers identified by participants. For example, learned teamwork skills may be used to navigate personality conflicts or the presence of negative emotions and ultimately lessen the impact of these barriers.

Emotions were also reported to be a key factor influencing teamwork in the OR. Each of these themes may be useful starting points to explore in moving toward enhanced teamwork education, training and guidelines. Addressing these considerations may also be a useful aid to teams during high acuity cases and call shifts, which were challenging times for teamwork identified by participants.

### Strengths and limitations

Although we achieved saturation, not all surgical specialties were represented, and most surgeons who participated practised general surgery. It will be important for future studies to determine whether there are variations in the themes reported here depending on the particular surgical specialty. Similarly, OR support staff, such as attendants, did not participate in the study and only two perfusionists participated. Consequently, this study cannot draw conclusions based on the experiences of OR professions outside of nursing, anaesthesia or surgery. Nevertheless, our study did include a balance of trainees and non-trainees and female and male healthcare professionals across these three professions. Most participants, however, were from an Ottawa-based hospital. Results may therefore not be representative of teamwork experiences in other hospitals or geographic locations. For example, there may be different norms and practices in different places related to power and hierarchy. These should be examined in future work.

Unlike other studies [20, 18], we did not aim to understand teamwork in one specialty, but teamwork in general. A key strength of our study is its large interprofessional sample, comprehensive and theory-informed interview guide, and conceptual generalizability and transferability [66]. Not only were we able to obtain important insights about teamwork from participants of different OR professions, but also, we were able to identify how numerous factors work together to shape barriers and enablers. This includes the finding that power and hierarchy in the OR exist along numerous social lines. It will be important for future work to further explore the larger social and structural factors influencing what is experienced as a barrier or enabler, by whom, and why. Although the TDF is useful for identifying specific influences on healthcare professional behaviour in order to inform intervention development, there may be other models, theories and frameworks that may be applied in future work to better understand broader cultural and contextual influences on teamwork and the inter-relationship between individual, team, and environmental factors. Nevertheless, this study reprsesnts a first step toward providing the type of data needed to move toward more effective interprofessional teamwork interventions for the OR.

## Conclusion

Our study identified key determinants of OR teamwork from an interprofessional perspective using a theoretically informed and systematic approach. Results suggest that achieving optimal teamwork in the OR may require a multi-level intervention that addresses individual, team and systems-level factors with particular attention to complex social and professional hierarchies.

## Supporting information

S1 Appendix Domains, definitions, and constructs of the Theoretical Domains Framework. (DOCX)

S2 Appendix Semi-structured interview guide for determining facilitators and barriers to effective teamwork in the OR. (DOCX)

S3 Appendix Themes and frequencies across professions for relevant and non-relevant TDF domains. (DOCX)
