# Supplementary material for: Barriers and enablers to effective interprofessional teamwork in the operating room: A qualitative study using the Theoretical Domains Framework
Source: PLoS One. 2021 Apr 22;16(4):e0249576. doi: 10.1371/journal.pone.0249576 (PMC8061974; doi:10.1371/journal.pone.0249576)
Supplement: S1 Appendix — (DOCX) [file pone.0249576.s001.docx]

# S1 Appendix. Domains, definitions, and constructs of the Theoretical Domains Framework*

| **Domain** | **Definition** | **Constructs** |
| --- | --- | --- |
| 1. Knowledge | An awareness of the existence of something | ﻿Knowledge (including knowledge of condition/scientific rationale) Procedural knowledge  Knowledge of task environment |
| 2, Skills | ﻿An ability or proficiency acquired through practice) | ﻿Skills  Skills development Competence Ability  Interpersonal skills  Practice Skill assessment  Professional |
| 3. Social/professional role and identity | ﻿A coherent set of behaviours and displayed personal qualities of an individual in a social or work setting | ﻿Professional identity  Professional role  Social identity  Identity  Professional boundaries Professional confidence  Group identity  Leadership  Organisational commitment  Self-confidence |
| 4. Beliefs about capabilities | ﻿Acceptance of the truth, reality or validity about an ability, talent or facility that a person can put to constructive use | ﻿Self-confidence  Perceived competence  Self-efficacy  Perceived behavioural control Beliefs  Self-esteem  Empowerment  Professional confidence |
| 5. Optimism | ﻿The confidence that things will happen for the best or that desired goals will be attained | ﻿Optimism  Pessimism  Unrealistic optimism |
| 6. Beliefs about consequences | ﻿Acceptance of the truth, reality, or validity about outcomes of a behaviour in a given situation | ﻿Beliefs  Outcome expectancies Characteristics of outcome expectancies  Anticipated regret  Consequents |
| 7. Reinforcement | ﻿Increasing the probability of a response by arranging a dependent relationship, or contingency, between the response and a given stimulus | ﻿Rewards (proximal/distal, valued/not valued, probable/improbable)  Incentives  Punishment  Consequents  Reinforcement  Contingencies  Sanctions  Stability |
| 8. Intentions | ﻿A conscious decision to perform a behaviour or a resolve to act in a certain way | ﻿Stability of intentions  Stages of change model Transtheoretical model and stages of change |
| 9. Goals | ﻿Mental representations of outcomes or end states that an individual wants to achieve | ﻿Goals (distal/proximal)  Goal priority  Goal/target setting  Goals (autonomous/controlled) Action planning  Implementation intention |
| 10. Memory, attention and decision processes | ﻿The ability to retain information, focus selectively on aspects of the environment and choose between two or more alternatives | ﻿Memory Attention  Attention control  Decision making  Cognitive overload/tiredness |
| 11. Environmental context and resources | ﻿Any circumstance of a person’s situation or environment that discourages or encourages the development of skills and abilities, independence, social competence and adaptive behaviour | ﻿Environmental stressors Resources/material resources Organisational culture/climate Salient events/critical incidents Person × environment interaction Barriers and facilitators |
| 12. Social influences | ﻿Those interpersonal processes that can cause individuals to change their thoughts, feelings, or behaviours) | ﻿Social pressure  Social norms  Group conformity  Social comparisons  Group norms  Social support  Power  Intergroup conflict  Alienation  Group identity  Modelling |
| 13. Emotion | ﻿A complex reaction pattern, involving experiential, behavioural, and physiological elements, by which the individual attempts to deal with a personally significant matter or event | Fear  Anxiety  Affect  Stress  Depression  Positive/negative affect  Burnout |
| 14. Behavioral regulation | ﻿Anything aimed at managing or changing objectively observed or measured actions | ﻿Self-monitoring  Breaking habit  Action planning |

*Used with permission from Atkins et al. 2017 [26].
