# Supplementary material for: Barriers and enablers to effective interprofessional teamwork in the operating room: A qualitative study using the Theoretical Domains Framework
Source: PLoS One. 2021 Apr 22;16(4):e0249576. doi: 10.1371/journal.pone.0249576 (PMC8061974; doi:10.1371/journal.pone.0249576)
Supplement: S2 Appendix — (DOCX) [file pone.0249576.s002.docx]

# S2 Appendix. Semi-structured interview guide for determining facilitators and barriers to effective teamwork in the OR

**Preamble**

*The general aim of the interview is to help us understand your experiences with teamwork in the operating room. Interviews will be recorded, transcribed and anonymized. These transcripts will be qualitatively analyzed to help us understand behaviors associated with intraoperative teamwork.*

*We want to know what influences you when you are working with others in the operating room to practice teamwork skills. There are no right or wrong answers here; We are trying to understand how different clinicians approach this issue, so please answer frankly.*

*I’d like to start with some basic demographic questions:*

1. What is your professional role? [surgeon, nurse, anesthesiologist]

a. **IF SURGEON:** What is your surgical department?

2. How many years have you practiced [surgery/nursing/anesthesia]?

a. Are you a trainee? [medical/nursing student, resident, fellow]

3. What is your age?

4. How would you describe your race/ethnicity or cultural background?

5. How would you describe your gender identity?

*Thank you.*

*For the rest of the interview, I have some slightly more specific questions about what influences your teamwork practices. Some questions may seem repetitive, but please bear with me as the questions are derived from different theories of human behaviour and we are trying to figure out which theories best apply in this area.*

*Are you ready to get started?*

1. How would you define teamwork in your professional context? (*Prompt: What does teamwork mean to you?)*
   1. What makes an effective (or successful/good) team? Give me an example. *(Prompt: What behaviours or practices would you consider to be part of effective teamwork? For example, communication or cooperation.)*
   2. How would you describe poor teamwork performance? Give me an example.
   3. Do you think teamwork mean the same thing or something different to each OR profession (nursing, anesthesia, surgery)?
   4. What kinds of qualities or skills do you value in team members that you work with?
2. In your experience, how do individuals contribute to or impact the teamwork of the whole interprofessional team?
3. Are you aware of any existing best practices for effective teamwork in the operating room? *(Prompt: What are they/where have you heard of them)*
4. Is teamwork an automatic part of your job for every case or is it something you deliberately focus on in the OR?
   1. Is there anything that influences how much you think about or focus on teamwork in the OR? *(Prompt: for example, who is in the room, the type of case, etc.)*
5. Do you make a conscious effort to be a good “team player” during surgery?
   1. How easy or difficult is this for you?
   2. Do you think anything would happen if you were not a good “team player” during surgery? *(Prompt: to patients, to colleagues, yourself, short and long term)*
6. As a [profession], is there anything that influences your approach to teamwork in the OR? *(Prompt: your training, a protocol, an order set, presence of a trainee or anything else)*
7. Are there any aspects of the OR environment itself that influence teamwork for you and/or for your colleagues? (*Prompt: e.g. physical vs. personnel vs. resource factors*)
8. Is there anything else that might influence your ability to engage in teamwork?
   1. What would help you overcome these problems/difficulties? *(Prompt: skills training in medical curriculum, communication techniques, formal training programs, educational material on line or by mail)*
9. What do you think happens when OR teams practice effective teamwork? (*Prompt: to patients, to colleagues, yourself, short and long term)*
10. What do you think happens if OR teams do NOT practice effective teamwork? (*Prompt: to patients, to colleagues, yourself, short and long term)*
11. Considering other priorities that you may have, how important is good teamwork in the OR for you? (Prompt: Scale of 1 – least important to 10 – highest importance. Why?)
12. Is good teamwork in the OR something that you want? Why?
13. Is good teamwork something that you feel you need to practice? Why or why not?
14. Do your emotions ever influence whether or not you engage in good teamwork in the OR? Explain.
15. Are there emotional aspects of practicing teamwork skills? [if yes] What are they?
16. Does the type of shift influence your emotions and their effect on teamwork in the OR? Explain. (*Prompt: for example, on call vs. day time or emergency vs elective case*)
17. Would any other team member influence whether or not you engage in good teamwork in the OR? *(Prompt: who else? Other staff, trainees?)*
18. Are there any incentives that have worked for you in the past that help you to practice good teamwork in the OR? *(Prompt: What helps you follow through with good teamwork?)*
19. Is there anything that prevents you from following through with good teamwork?
20. What are some personal ways/steps of working that help you to practice good teamwork?
21. Do you make specific plans to practice good teamwork? *(Prompt: for example, if a certain situation arises in the OR))?*
22. If you wanted to implement changes in your own practice to perform better from a teamwork perspective, what do you think would be the steps necessary to do this?
23. Do you believe the effort associated with practicing good teamwork is worth the benefit? Why or why not?
24. Do you think your gender influences how you interact with others in the OR? *(If yes, in what ways? If no, why not?)*
25. Do you think the ways others interact with you in the OR are influenced by your gender? By their gender? *(Why/why not? How?)*
26. Are there are any factors related to your social position (for example age, language or accent, trainee status, etc.) that you feel influences your ability to engage in good teamwork?
    1. In what way might these factors promote or impede good teamwork?
27. Is there anything else you would like to add about teamwork in the OR that we haven’t touched upon yet in this interview?
