# Supplementary material for: Barriers and enablers to effective interprofessional teamwork in the operating room: A qualitative study using the Theoretical Domains Framework
Source: PLoS One. 2021 Apr 22;16(4):e0249576. doi: 10.1371/journal.pone.0249576 (PMC8061974; doi:10.1371/journal.pone.0249576)
Supplement: S3 Appendix — (DOCX) [file pone.0249576.s003.docx]

# S3 Appendix. Themes and frequencies across professions for relevant and non-relevant TDF domains

| **DOMAIN** | **Total across professions (n=66)** | **RNs/RPNs**  **(n=21)** | **Anaesthesiologists (n=17)** | **Surgeons (n=26)** | **Perfusionists (n=2)** |
| --- | --- | --- | --- | --- | --- |
| **Theme** |  |  |  |  |  |
| *Sub-theme* |  |  |  |  |  |
| **BEHAVIORAL REGULATION** | **-** | **-** | **-** | **-** | **-** |
| **1. Communication Practices** | 47 | 16 | 12 | 18 | 1 |
| *1a. Ask questions* | 4 | 4 | 0 | 0 | 0 |
| *1b. Communicate clearly* | 15 | 5 | 4 | 5 | 1 |
| *1c. Existing tools as opportunity to communicate* | 4 | 2 | 1 | 1 | 0 |
| *1d. Express concerns in advance* | 6 | 0 | 5 | 1 | 0 |
| *1e. Explain actions out loud* | 2 | 0 | 0 | 2 | 0 |
| *1f. Identify critical points* | 1 | 0 | 0 | 1 | 0 |
| *1g. Include all team members in communications* | 2 | 1 | 0 | 1 | 0 |
| *1h. Intentional use of language* | 2 | 1 | 1 | 0 | 0 |
| *1i. Need for communication template* | 1 | 0 | 0 | 1 | 0 |
| *1j. Pause* | 1 | 0 | 0 | 1 | 0 |
| *1k. Speak loudly* | 1 | 0 | 0 | 1 | 0 |
| *1l. Speak up* | 8 | 3 | 1 | 4 | 0 |
| **2. Feedback** | 12 | 1 | 6 | 4 | 1 |
| *2a. Debriefing on case* | 5 | 0 | 2 | 2 | 1 |
| *2b. Feedback on performance* | 7 | 1 | 4 | 2 | 0 |
| **3. Plan or Prepare** | 11 | 5 | 1 | 5 | 0 |
| *3a. Plan for the day* | 8 | 4 | 0 | 4 | 0 |
| *3b. Prioritize* | 1 | 0 | 1 | 0 | 0 |
| *3c. Team preparation* | 2 | 1 | 0 | 1 | 0 |
| **4. Helping Others** | 0 | 1 | 0 | 3 | 0 |
| *4a. Put own responsibilities aside to help team when needed* | 0 | 0 | 0 | 3 | 0 |
| *4b. Team up to speak up* | 0 | 1 | 0 | 0 | 0 |
| **5. Self-management** | 12 | 0 | 4 | 8 | 0 |
| *5a. Be aware of how your behaviour affects others* | 4 | 0 | 2 | 2 | 0 |
| *5b. Be open-minded* | 1 | 0 | 0 | 1 | 0 |
| *5c. Humanize oneself* | 2 | 0 | 0 | 2 | 0 |
| *5d. Re-frame the situation* | 5 | 0 | 2 | 3 | 0 |

|  | **Total across professions (n=66)** | **RNs/RPNs**  **(n=21)** | **Anaesthesiologists (n=17)** | **Surgeons (n=26)** | **Perfusionists (n=2)** |
| --- | --- | --- | --- | --- | --- |
| **BEHAVIORAL REGULATION (continued)** | - | - | - | - | - |
| **6. People management** | 65 | 10 | 17 | 26 | - |
| *6a. Be nice or polite to others* | 7 | 3 | 2 | 1 | 0 |
| *6c. Confrontation/conflict management* | 7 | 1 | 1 | 3 | 0 |
| *6d. Get to know others/build relationships* | 7 | 0 | 2 | 3 | 0 |
| *6e. Make others feel included/valued* | 7 | 2 | 2 | 2 | 0 |
| *6g. Pick your team* | 3 | 0 | 0 | 3 | 0 |
| *6h. Tailor interactions with others to the situation* | 12 | 3 | 3 | 4 | 0 |
| *6i. Understand others’ roles* | 6 | 0 | 2 | 3 | 0 |
| *6j. Use/learn names* | 16 | 1 | 5 | 7 | 0 |
| **BELIEFS ABOUT CONSEQUENCES** | **-** | **-** | **-** | **-** | **-** |
| **1. Good teamwork results in an efficient OR** | 39 | 14 | 8 | 16 | 1 |
| **2. Good teamwork facilitates individual performance** | 2 | 1 | 0 | 1 | 0 |
| **3. Bad teamwork inhibits individual performance** | 5 | 1 | 2 | 2 | 0 |
| **4. Good teamwork improves HCP occupational well-being** | 53 | 16 | 14 | 21 | 2 |
| **5. Good teamwork improves patient safety/outcomes in the OR** | 42 | 14 | 10 | 16 | 2 |

|  | **Total across professions (n=66)** | **RNs/RPNs**  **(n=21)** | **Anaesthesiologists (n=17)** | **Surgeons (n=26)** | **Perfusionists (n=2)** |
| --- | --- | --- | --- | --- | --- |
| **EMOTIONS** | **-** | **-** | **-** | **-** | **-** |
| **1. Negative emotions interfere with effective teamwork** | 49 | 15 | 14 | 19 | 1 |
| **2. Emotions belong outside of the OR** | 13 | 6 | 1 | 6 | 0 |
| **3. Compassion and empathy are necessary to be a good team member** | 1 | 0 | 0 | 1 | 0 |
| **4. Others can influence my emotions, and as a result, teamwork** | 16 | 8 | 4 | 3 | 1 |
| **5. Experience mitigates the negative effects of emotions on teamwork** | 3 | 1 | 0 | 2 | 0 |
| **6. Positive emotions promote effective teamwork** | 6 | 2 | 1 | 3 | 0 |
| **7. Intuition is part of good practice** | 1 | 0 | 1 | 0 | 0 |
| **ENVIRONMENTAL CONTEXT AND RESOURCES** | **-** | **-** | **-** | **-** | **-** |
| **1. Resource-related challenges (e.g. staffing, equipment, time)** | 53 | 23 | 8 | 22 | 0 |
| **2. The presence of trainees** | 26 | 8 | 6 | 11 | 1 |
| *2a. Trainees can inhibit teamwork* | 16 | 6 | 3 | 7 | 0 |
| *2b. Trainees can positively affect teamwork* | 8 | 2 | 2 | 3 | 1 |
| *2c. Trainees do not affect teamwork* | 2 | 0 | 1 | 1 | 0 |
| **3. Different pay structures by professional group** | 4 | 0 | 0 | 4 | 0 |
| **4. Distractions** | 16 | 4 | 3 | 8 | 1 |
| **5. Physical environment (e.g. hospital layout, OR room)** | 26 | 6 | 5 | 14 | 1 |
| **6. Severity or type of case** | 40 | 5 | 12 | 23 | 0 |
| **7. On-call or night shift** | 34 | 8 | 10 | 15 | 1 |
| **8. High-stakes environment** | 5 | 3 | 1 | 1 | 0 |

|  | **Total across professions (n=66)** | **RNs/RPNs**  **(n=21)** | **Anaesthesiologists (n=17)** | **Surgeons (n=26)** | **Perfusionists (n=2)** |
| --- | --- | --- | --- | --- | --- |
| **ENVIRONMENTAL CONTEXT AND RESOURCES (continued)** | - | - | - | - | - |
| **9. Team climate** | 23 | 6 | 7 | 9 | 1 |
| **10. Organizational culture** | 19 | 7 | 2 | 9 | 1 |
| *10a. Disconnect between administration and frontline staff* | 6 | 2 | 1 | 3 | 0 |
| *10b. Hospital protocols or practices* | 8 | 2 | 1 | 5 | 0 |
| *10c. Perceptions of senior management* | 4 | 3 | 0 | 0 | 1 |
| *10d. Perceptions of system vs. patient first* | 1 | 0 | 0 | 1 | 0 |
| **14. Familiarity with specific service or procedure** | 7 | 2 | 2 | 3 | 0 |
| **15. Number of people in the room** | 6 | 4 | 1 | 1 | 0 |
| **16. No impact** | 6 | 3 | 0 | 3 | 0 |
| **GOALS** | **-** | **-** | **-** | **-** | **-** |
| **1. Teamwork is important to achieve the best care for the patient.** | 33 | 10 | 8 | 14 | 1 |
| **2. Teamwork is necessary for me to be able to do my job effectively.** | 11 | 6 | 2 | 3 | 0 |
| **3. Teamwork is important but not as important as other things.** | 12 | 1 | 3 | 7 | 1 |
| **4. Teamwork facilitates efficiency, a top priority.** | 3 | 0 | 1 | 2 | 0 |
| **INTENTIONS** | **-** | **-** | **-** | **-** | **-** |
| **1. I generally make a conscious effort to engage in good teamwork.** | 36 | 10 | 12 | 13 | 1 |
| **KNOWLEDGE** | **-** | **-** | **-** | **-** | **-** |
| **1. Lack of awareness regarding best practices** | 39 | 13 | 10 | 14 | 2 |
| **2. Definition of teamwork is working toward a common goal** | 54 | 14 | 15 | 24 | 1 |
| **3. Knowledge of tools or practices that support teamwork** | 24 | 5 | 5 | 13 | 1 |
| **4. Teamwork is a “buzzword” but not well understood** | 3 | 1 | 0 | 1 | 1 |
| **MEMORY, ATTENTION AND DECISION PROCESSES** | **-** | **-** | **-** | **-** | **-** |
| **1. Teamwork is both an automatic and deliberate process** | 10 | 5 | 1 | 4 | 0 |
| **2. Teamwork becomes automatic with experience** | 6 | 1 | 1 | 4 | 0 |
| **3. Teamwork is a deliberate action** | 4 | 0 | 0 | 4 | 0 |

|  | **Total across professions (n=66)** | **RNs/RPNs**  **(n=21)** | **Anaesthesiologists (n=17)** | **Surgeons (n=26)** | **Perfusionists (n=2)** |
| --- | --- | --- | --- | --- | --- |
| **MEMORY, ATTENTION AND DECISION PROCESSES (continued)** | - | - | - | - | - |
| **4. Teamwork is natural or innate** | 14 | 8 | 3 | 3 | 0 |
| **REINFORCEMENT** | **-** | **-** | **-** | **-** | **-** |
| **1. Personal rewards or benefits** | 22 | 6 | 5 | 10 | 1 |
| *1a. Chance for a break* | 1 | 0 | 1 | 0 | 0 |
| *1b. Finish case faster* | 7 | 1 | 1 | 5 | 0 |
| *1c. Good relationships* | 2 | 0 | 0 | 1 | 1 |
| *1d. Opportunities in the future* | 1 | 0 | 0 | 1 | 0 |
| *1e. Recognition* | 11 | 5 | 3 | 3 | 0 |
| **2. No incentives** | 5 | 2 | 1 | 2 | 0 |
| **3. Good patient care** | 3 | 1 | 1 | 1 | 0 |
| **4. Evidence** | 1 | 0 | 1 | 0 | 0 |
| **SKILLS** | **-** | **-** | **-** | **-** | **-** |
| **1. Teamwork is a skill** | 5 | 1 | 2 | 2 | 0 |
| **2. Teamwork skills improve with experience** | 5 | 2 | 2 | 1 | 0 |
| **3. Teamwork skills are often learned on the job** | 9 | 4 | 1 | 4 | 0 |
| **4. Teamwork skills are distinct from technical skills** | 6 | 3 | 1 | 1 | 1 |
| **5. Teamwork skills are not part of training** | 12 | 3 | 1 | 6 | 2 |
| **6. Good teamwork takes practice** | 20 | 8 | 5 | 5 | 2 |
| **7. Some teamwork skills obtained from past simulation training** | 3 | 0 | 3 | 0 | 0 |
| **SOCIAL INFLUENCES** | **-** | **-** | **-** | **-** | **-** |
| **1. Familiarity with team members** | 38 | 9 | 10 | 18 | 1 |
| **2. Interprofessional hierarchy** | 16 | 5 | 5 | 6 | 0 |
| **3. Gendered hierarchy** | 31 | 9 | 6 | 14 | 2 |
| **4. Experience hierarchy** | 7 | 1 | 2 | 4 | 0 |
| **5. Impact of others’ personalities** | 60 | 20 | 14 | 24 | 2 |
| **6. Others’ perceptions** | 3 | 0 | 1 | 2 | 0 |
| **7. Clear leadership** | 13 | 2 | 3 | 8 | 0 |

|  | **Total across professions (n=66)** | **RNs/RPNs**  **(n=21)** | **Anaesthesiologists (n=17)** | **Surgeons (n=26)** | **Perfusionists (n=2)** |
| --- | --- | --- | --- | --- | --- |
| **SOCIAL INFLUENCES (continued)** | - | - | - | - | - |
| **8. Anaesthesiologist-surgeon relationship** | 5 | 1 | 2 | 2 | 0 |
| **SOCIAL/PROFESSIONAL ROLE AND IDENTITY** | **-** | **-** | **-** | **-** | **-** |
| **1. Gender** | 59 | 19 | 13 | 25 | 2 |
| **2. Level of experience** | 16 | 3 | 2 | 11 | 0 |
| **3. Age** | 0 | 0 | 0 | 0 | 0 |
| **4. Intersection of multiple identity characteristics** | 26 | 6 | 8 | 11 | 1 |
| **5. Country of medical training** | 4 | 1 | 2 | 1 | 0 |
| **6. Roles or experience outside of clinical OR practice** | 6 | 2 | 2 | 2 | 0 |
| **7. Language or accent** | 13 | 8 | 2 | 2 | 1 |
| **8. Ethnicity or cultural background** | 8 | 5 | 0 | 3 | 0 |
| **9. Teamwork is part of professional role** | 36 | 12 | 10 | 13 | 1 |
| **10. Personality** | 10 | 5 | 3 | 2 | 0 |
| **11. Different professional norms/values/goals/perceptions** | 37 | 7 | 10 | 18 | 2 |
